# Effect of polydeoxyribonucleotide on early bone formation in lateral bone augmentation with immediate implant placement: an experimental in vivo study

**DOI:** 10.1038/s41598-023-43213-8

**Published:** 2023-10-06

**Authors:** Dongseob Lee, Jungwon Lee, Yang-Jo Seol, Yong-Moo Lee, Ki-Tae Koo

**Affiliations:** 1 Department of Periodontology, School of Dentistry and Dental Research Institute, Seoul National University and Seoul National University Dental Hospital, 101, Daehak-ro, Jongno-gu, Seoul, 03080 Republic of Korea; 2 National Dental Care Center for Persons with Special Needs, Seoul National University Dental Hospital, Seoul, 03080 Republic of Korea; 3 One-Stop Specialty Center, Seoul National University Dental Hospital, Seoul, 03080 Republic of Korea; 4 Department of Periodontology, Seoul National University Dental Hospital, Seoul, 03080 Republic of Korea

**Keywords:** Biotechnology, Drug discovery, Medical research

## Abstract

This study investigated early bone formation using collagenated biphasic calcium phosphate (CBCP) with or without polynucleotide (PDRN). Third (P3) or fourth (P4) premolars of six male beagle dogs were extracted and 5-mm-high dehiscence defects were created, followed by 3D-printed implant placement. The buccal bone defects were grafted with (i) CBCP and collagen membrane or (ii) CBCP soaked in polydeoxyribonucleotide (CBCP/PDRN) and collagen membrane. Samples of the experimental sites were harvested at 2- and 6-weeks post-surgery. The specimens were evaluated with radiologic and histomorphometric analysis. No significant differences were found between the CBCP and CBCP/PDRN groups in the micro-CT analysis at 2 or 6 weeks. No significant differences were observed in bone-to-implant contact (BIC) or bone area fraction occupancy (BAFO) in buccal augmented and lingual non-augmented areas. In the qualitative analysis, the new bone (NB) area and NB proportion in buccal augmented areas showed significantly higher values in the CBCP/PDRN group than in the CBCP group at 2 and 6 weeks. Peri-implant buccal dehiscence defects with immediate 3D-printed implant placement were corrected using a collagen membrane and CBCP or CBCP/PDRN. PDRN might have the potential to facilitate early bone formation with sufficient stability over time in dehiscence defects.

## Introduction

The concept of placing the implant in a location with a sufficient amount of bone has shifted to prosthetically driven implant placement to achieve esthetically favorable prostheses^1^. However, bone defects, such as dehiscence or fenestration, can occur in atrophic alveolar ridges, which may exposure the implant fixture. To ensure long-term survival and esthetic outcomes for dental implants, it is necessary to perform lateral bone augmentation using bone substitutes to cover the implant fixture. A recent systematic review has demonstrated that lateral bone augmentation with bone graft material, performed concomitantly with implant placement, can be a successful treatment modality for correcting deficient bone^2^.

The ability of various bioactive agents to facilitate bone formation in the early stages following bone augmentation has been investigated. Bone morphogenetic proteins (BMPs) have been the most frequently studied agents in the field of bone regeneration^3^. Recombinant human bone morphogenetic protein 2 (rhBMP-2) has been approved for sinus floor elevation and ridge preservation^3,4^. Despite its osteoinductive potency, many clinicians are still reluctant to apply rhBMP-2 for bone augmentation due to complications including swelling, seroma formation, and cancer^5^.

Polydeoxyribonucleotide (PDRN) is a substance extracted from the sperm cells of *Oncorhynchus mykiss* or *Oncorhynchus keta*^6^ that stimulates angiogenesis by activating purinergic A2A receptors^7^, thereby causing cell proliferation and promoting wound healing^8,9^. Angiogenesis plays a pivotal role in early bone formation^10^, and several studies investigating the promotion of early osteogenesis using PDRN, which promotes angiogenesis, have recently been conducted^11–13^. In a preclinical study, we also reported that PDRN might enhance soft tissue healing^14^ and early bone formation in maxillary sinus elevation^15^.

The use of collagenated block bone to restore bone volume has been investigated in preclinical and clinical studies^16–20^. Adding collagen to bone substitutes improved their stability and reduced the displacement of bone graft material during flap closure. Furthermore, several studies have demonstrated the potential of collagenated block bone to serve as a carrier for various growth factors or bioactive agents, such as collagen matrix^21–23^. In this context, it would be promising to investigate the use of collagenated block bone in lateral augmentation in peri-implant bone defects with various growth factors.

The aim of the present study was to investigate the effects of PDRN on early bone formation in vivo after lateral bone augmentation with simultaneous implant placement.

## Materials and methods

### Sample size calculation

A previous study reported bone-to-implant contact of 19.57 ± 10.16% when immediate implant placement was performed concomitantly with a bone graft using bone substitutes^24^. In our study, we considered a mean difference of over 15% between the two groups as statistically significant, with the standard deviation set to 8%. We calculated that six animals would be necessary, with α set to 0.05 and β set to 0.8 (effect size: 1.875).

### Study animals and ethical statement

Six male beagle dogs, aged about 1 year and weighing 9 to 13 kg, were included. The Institutional Animal Care and Use Committee of Seoul National University approved this animal study (No. SNU-220216-1-2), which complied with the Animal Research, Reporting of In Vivo Experiment (ARRIVE) guidelines^25^. The experiment was carried out according to the guideline and regulations of Institutional Animal Care and Use Committee of Seoul National University^26^.

### 3D-printed implants

3D-printed implants were fabricated using a direct metal laser sintering machine with Ti–6Al–4V powder. This process was conducted with Dentium Build Processor 1.4.7 (Dentium, Seoul, Korea) powered by KETI Slicing Engine. 3D-printed implants underwent Large-grit sandblasting and acid-etching (SLA) surface treatment. Finally, gamma-ray irradiation, emitting short wavelength light from a cobalt-60 radioactive isotope, was performed to sterilize all the 3D-printed implants.

### Study design

**Figure 1:**
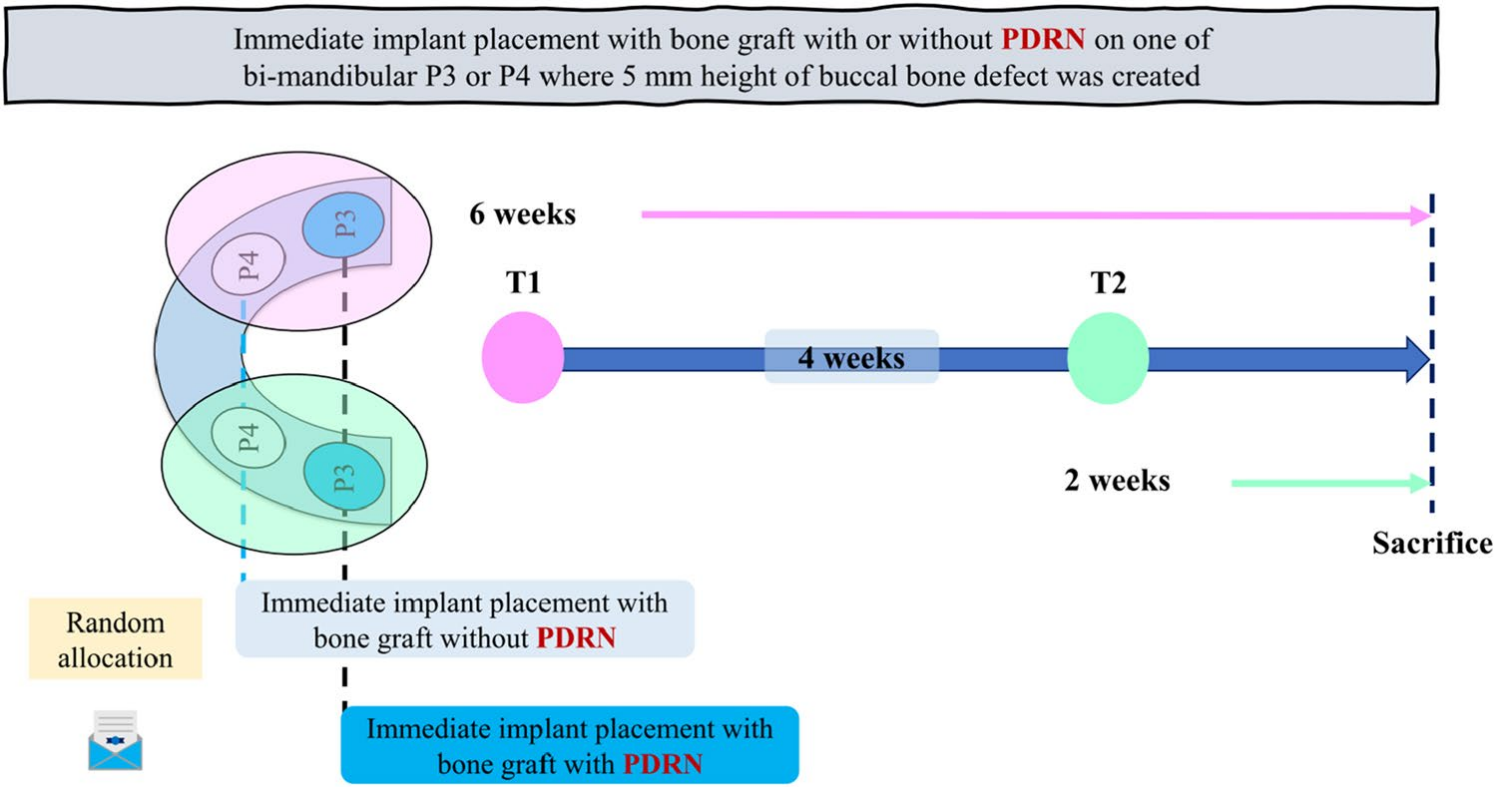
Time frame and experimental design of this study. Immediate implant placement and a bone graft were performed at the third premolar, fourth premolar (P3 or P4) on side of mandible. 4 weeks following the surgical procedure, the same surgical intervention was performed on the other side of the mandible.

The timeframe and experimental design of this study are presented in Fig. 1. The surgery was performed two times to undergo 2- and 6-weeks healing periods. Third premolar (P3) and fourth premolar (P4) at one side of mandible was extracted and followed by creating 5-mm-high buccal bone defect on each extracted site. 3D-printed implants were placed on the mesial root sites and bone graft was performed to cover buccal dehiscence. Bone graft was allocated randomly to one of the following groups: (i) CBCP with PDRN (test group) or (ii) CBCP without PDRN (control group). The random allocation was made with an Excel random number table. The same surgical procedure was performed on another mandible 4 weeks after the first surgery. All animals were euthanized after 2 weeks following the second surgery.

### Surgical procedures

**Figure 2:**
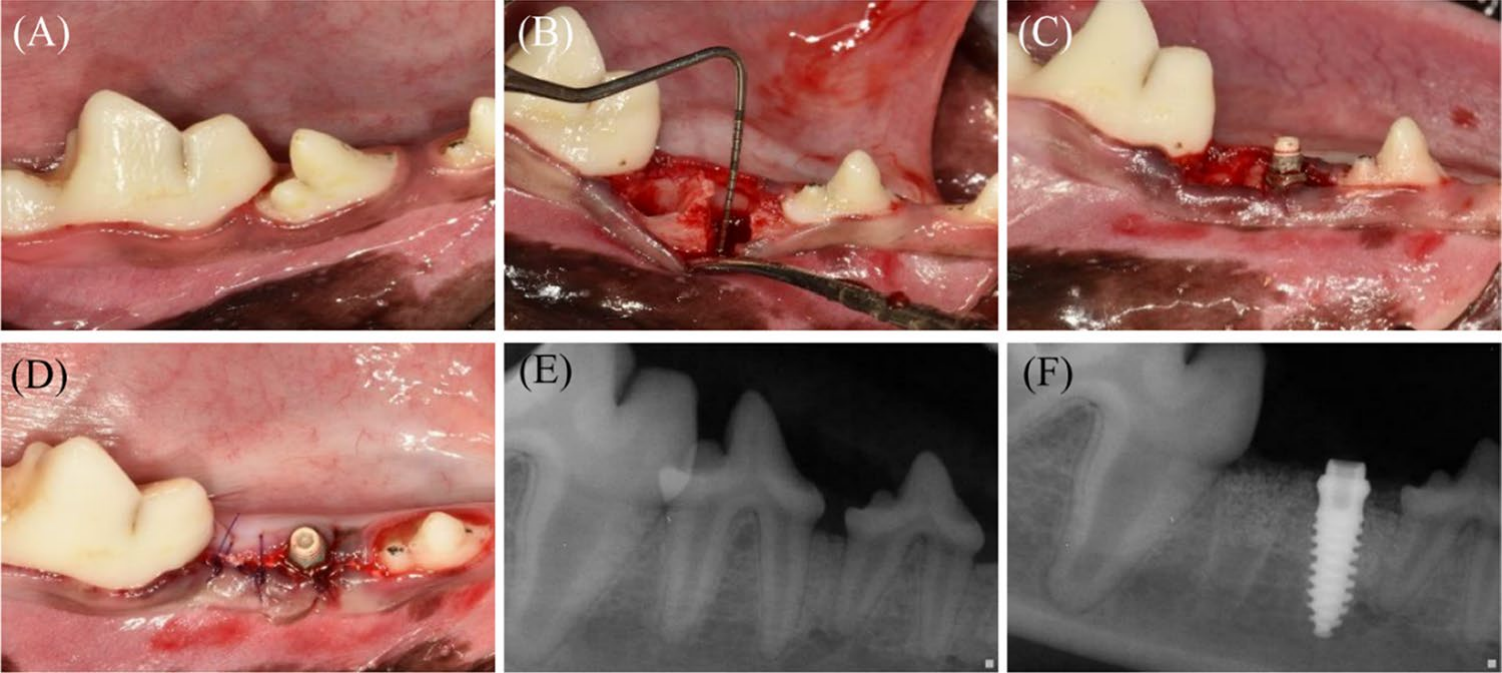
Clinical photographs and periapical radiographs. (**A**) Clinical photograph before the surgical procedure (**B**–**D**) Clinical photograph during surgery. After tooth extraction (bi-mandibular P4), 5-mm-high defects were created, and implants were placed with a simultaneous bone graft. (**E**) Periapical radiograph before surgery. (**F**) Periapical radiograph after surgery.

For the surgical procedure, each animal underwent general anesthesia via an intravenous injection of xylazine hydrochloride (3.5 mg/kg) (Rompun, Bayer Korea, Korea) and tiletamine/zolazepam (7.5 mg/kg) (Zoletil 50, Virbac, France). Local anesthesia was applied using 2% lidocaine HCl (1:100,000 epinephrine) (lidocaine HCl, Huons, Korea) before the surgical procedure. One of the selected teeth (P3 or P4) was hemisected using a diamond bur, followed by tooth extraction. A buccal flap on the mesial root site was elevated, creating 5-mm-high buccal bone defects with a rotary instrument. 3D-printed implants (Ф3.5 mm, 9 mm length, Dentium, Korea) were placed on the experimental, site and the buccal bone defects were grafted with CBCP (OSTEON 3 collagen, 6 × 5 mm; GENOSS, Korea) only or CBCP soaked in polydeoxyribonucleotide (5.625 mg/3 mL, GENOSS, Korea) for 10 min, as in a previous study^15^. A crosslinked collagen membrane (Collagen membrane-P, GENOSS, Korea) was applied to the grafted site, followed by suturing the flap with 5/0 Monosyn. After surgical procedure, all animals were injected with intravenous antibiotics (cefazolin injection, 15 mg/kg, Chongkundang, Korea) and analgesics (Metacam, 0.2 mg/kg, Boehringer Ingelheim, Germany). The surgical procedures are demonstrated in Fig. 2.

### Micro-computed tomography analysis

Block biopsies including the experimental sites were obtained 6 weeks after the first surgery. Prior to histological preparation, micro-computed tomography (micro-CT) data of the samples were obtained, using the same conditions as in a previous study (13.3-μm resolution, 60 kV energy, and 167 μA intensity using 0.5-mm aluminum filter)^27^. The scanned images were reconstructed using software (DataViewer 1.5.2.4 64-bit version, Bruker microCT, Skyscan, Kontich, Belgium) and the data were analyzed with CTAn software (Bruker-CT, Kontich, Belgium). In accordance with previous studies^27,28^, 8-bit grayscale values ranging from 30 to 80 was set to identify bone tissue. Bone volume/tissue volume (BV/TV), bone surface/bone volume (BS/BV), the trabecular bone pattern factor (Tb.Pf), and structure model index (SMI) values within the VOI were calculated.

### Histologic processing

All samples were dehydrated and embedded into methylmethacrylate resin block. Each block was cut with a diamond cutter (Exakt, Apparatebau, Germany). Histologic sections were made in the bucco-lingual direction. The sections were made to be about 100 μm thick, and the specimens were ground to 30-μm thickness with a diamond grinder. Finally, all specimens were stained with Goldner’s trichrome. All histologic images were scanned to obtain digital images, which were used to perform histomorphometric analysis.

### Histomorphometric analysis

New bone (NB), residual bone graft (RBG), and fibrovascular connective tissue (FVCT) were identified CaseViewer 2.2 software (3DHISTECH Ltd., Budapest, Hungary). The bone-to implant contact (BIC) and bone area fraction occupancy (BAFO) was calculated in the lingual non-augmented areas and buccal augmented areas, respectively. Additionally, a qualitative analysis was performed using measurements of new bone (NB), residual bone graft (RBG), and fibrovascular connective tissue (FVCT) in the buccal augmented area using ImageJ software (National Institutes of Health, Bethesda, MD, USA).

The following measurements were conducted in the buccal augmented area:

- Total augmented area (TAA): the augmented tissue area, including RBG, NB, and FVCT.
- Upper augmented area (UAA): the augmented tissue area beyond a horizontal line perpendicular to the implant long axis at the bottom of the imaginary buccal bone defect.
- Lower augmented area (LAA): the augmented tissue area below a horizontal line perpendicular to the implant long axis at the bottom of the imaginary buccal bone defect.
- Dislocation rate: LAA/TAA.

### Statistical analysis

All data on outcomes in the two groups are presented as means ± standard deviations. The Shapiro–Wilk test was performed to determine whether the data fit a normal distribution. The independent t-test or one-way analysis of variance with the Tukey multiple-comparison test was performed for the micro-CT and histomorphometric analyses at each time point. Statistical significance was set at p < 0.05.

## Results

### Clinical observations

All 24 experimental sites healed uneventfully without any signs of infection or inflammation. The peri-implant soft tissue was clinically healthy with a pink color.

### Micro-CT analysis

**Figure 3:**
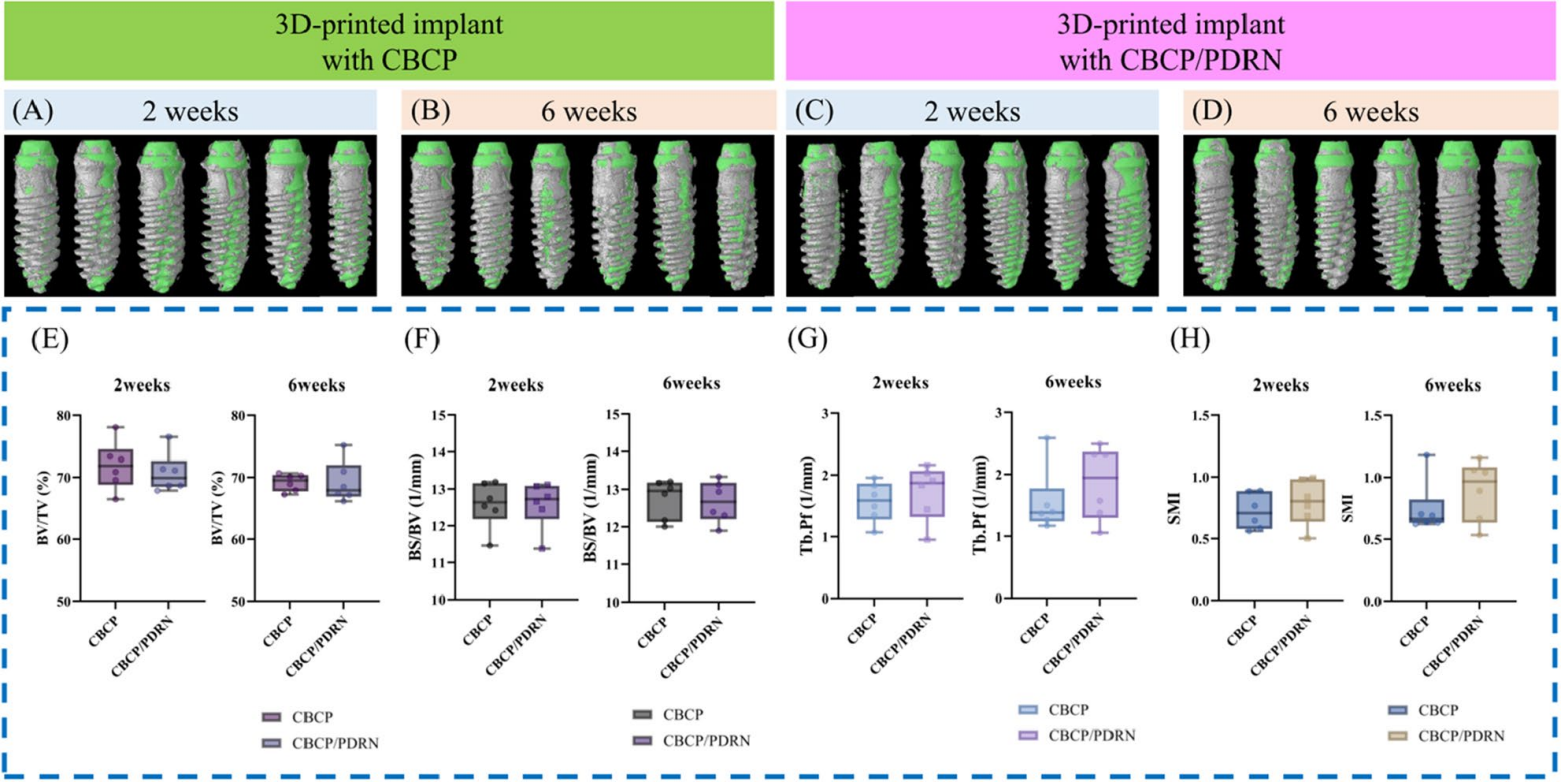
The outcomes of micro-CT analysis. (**A**–**D**) Gray and green areas indicate bone volume and implants in the volume of interest. (**E**–**H**) Comparable values in the CBCP and CBCP/PDRN groups were shown, without statistically significant differences for any parameters, including bone volume/tissue volume (BV/TV), bone surface/bone volume (BS/TV), trabecular bone pattern factor (Tb.Pf), and structure model index (SMI).

The outcomes of the micro-CT analysis in the CBCP and CBCP/PDRN groups are shown in Fig. 3. For all parameters analyzed in this study, comparable values for CBCP and CBCP/PDRN were observed, with no statistically significant differences. The BV/TV values of CBCP and CBCP/PDRN at 2 weeks were 71.88 ± 3.91% and 70.71 ± 3.18%, respectively. The BV/TV values of CBCP and CBCP/PDRN at 6 weeks were similar to those at 2 weeks (69.21 ± 1.39% and 69.23 ± 3.33%, respectively). The BS/TV values of CBCP and CBCP/PDRN at 2 weeks were 12.58 ± 0.63 mm^−1^ and 12.58 ± 0.64 mm^−1^, respectively. The values of CBCP and CBCP/PDRN at 6 weeks showed no significant changes compared with the values at 2 weeks (12.75 ± 0.52 mm^−1^ and 12.67 ± 0.55 mm^−1^, respectively). The SMI of CBCP and CBCP/PDRN at 2 weeks was 0.72 ± 0.15 mm^−1^ and 0.79 ± 0.19 mm^−1^, respectively. At 6 weeks, the SMI of CBCP and CBCP/PDRN was 0.74 ± 0.21 mm^−1^ and 0.89 ± 0.24 mm^−1^. No significant differences were found between the two groups at 2 or 6 weeks.

### Histologic observations

**Figure 4:**
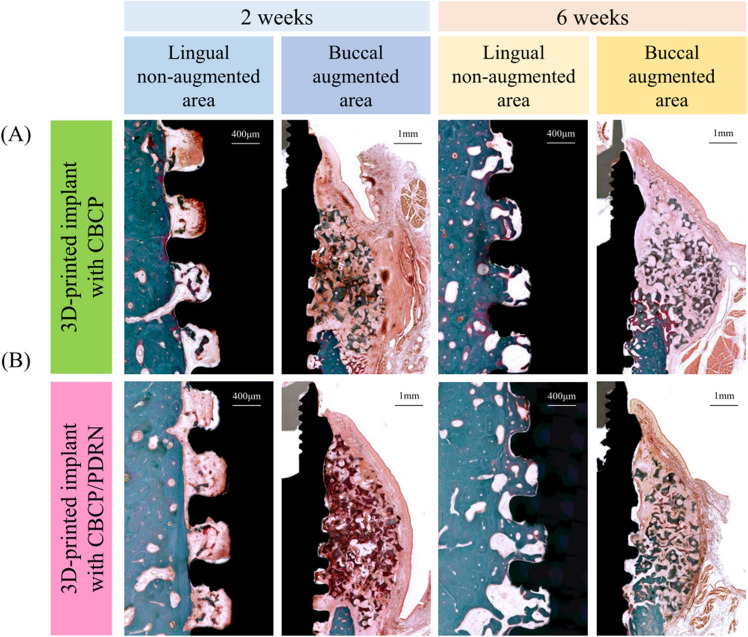
Representative histologic images in the lingual non-augmented and buccal augmented area. (**A**) 3D-printed implant with CBCP at 2 and 6 weeks and (**B**) 3D-printed implant with CBCP/PDRN at 2 and 6 weeks.

In the lingual non-augmented area, gradual new bone formation at the 3D-printed implant interface was observed in specimens at 2 and 6 weeks (Fig. 4). Time-dependent increases in bone mineralization and bone-to-implant contact were observed. Osteoid matrix indicating dynamic new bone formation was observed at 2 weeks of healing, whereas plexiform bone mixed with woven and lamella bone around the implant surface was found at 6 weeks of healing.

In the buccal augmented area, a time-dependent pattern of new bone formation was observed; however, the amount of new bone was less than in the lingual non-augmented area (Fig. 4). Most of the residual bone graft material was found in the artificially created dehiscence defects, although some bone graft material was observed below the defect sites.

### Histomorphometric analysis

#### BIC in the lingual non-augmented and buccal augmented areas

**Figure 5:**
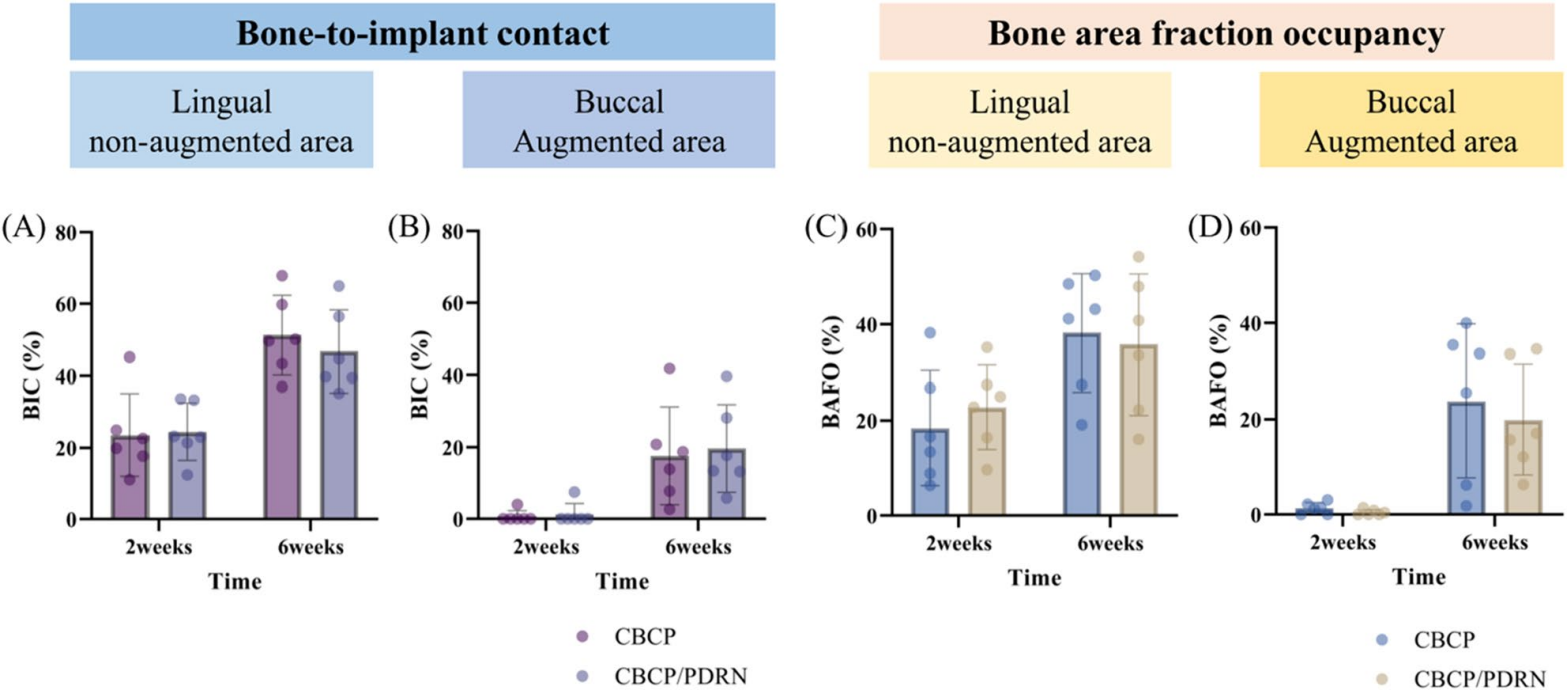
Outcomes of the histomorphometric analysis. (**A**,**B**) Bone-to-implant contact (BIC) and (**C**,**D**) bone area fraction occupancy (BAFO) in the lingual non-augmented and buccal augmented areas at 2 and 6 weeks.

The results for BIC in the lingual non-augmented and buccal augmented areas are presented in Fig. 5.

In the lingual area, the BIC values at 2 weeks were 23.58 ± 11.58% in the CBCP group and 24.53 ± 8.04% in the CBCP/PDRN group (p = 0.8729). The BIC values at 6 weeks were 51.31 ± 11.08% in the CBCP group and 46.78 ± 11.51% in the CBCP/PDRN group (p = 0.5037). No significant differences between the two groups were found at the 2- and 6-week time points.

In the augmented area, the BIC values at 2 weeks were 0.68 ± 1.63% in the CBCP group and 1.25 ± 3.05% in the CBCP/PDRN group (p = 0.8485). The BIC values at 6 weeks were 17.63 ± 13.65% in the CBCP group and 19.69 ± 12.22% in the CBCP/PDRN group (p = 0.7887). No significant differences between the two groups were noted at the 2- and 6-week time points.

#### BAFO in the lingual and augmented areas

The outcomes of BAFO in the lingual non-augmented and buccal augmented areas are presented in Fig. 5.

In the lingual area, the BAFO values at 2 weeks were 18.39 ± 12.06% in the CBCP group and 22.77 ± 8.87% in the CBCP/PDRN group (p = 0.4895). The BAFO values at 6 weeks were 38.28 ± 12.35% in the CBCP group and 36.81 ± 14.75% in the CBCP/PDRN group (p = 0.7592). No significant differences were noted between the two groups at the 2- and 6-week time points.

In the augmented area, the BAFO values at 2 weeks were 1.32 ± 1.21% in the CBCP group and 0.45 ± 0.59% in the CBCP/PDRN group (p = 0.1448). The BAFO values at 6 weeks were 23.76 ± 16.05% in the CBCP group and 19.89 ± 11.58% in the CBCP/PDRN group (p = 0.6425). No significant differences were noted between the two groups at the 2- and 6-week time points.

#### Dislocation rate in the augmented area

**Figure 6:**
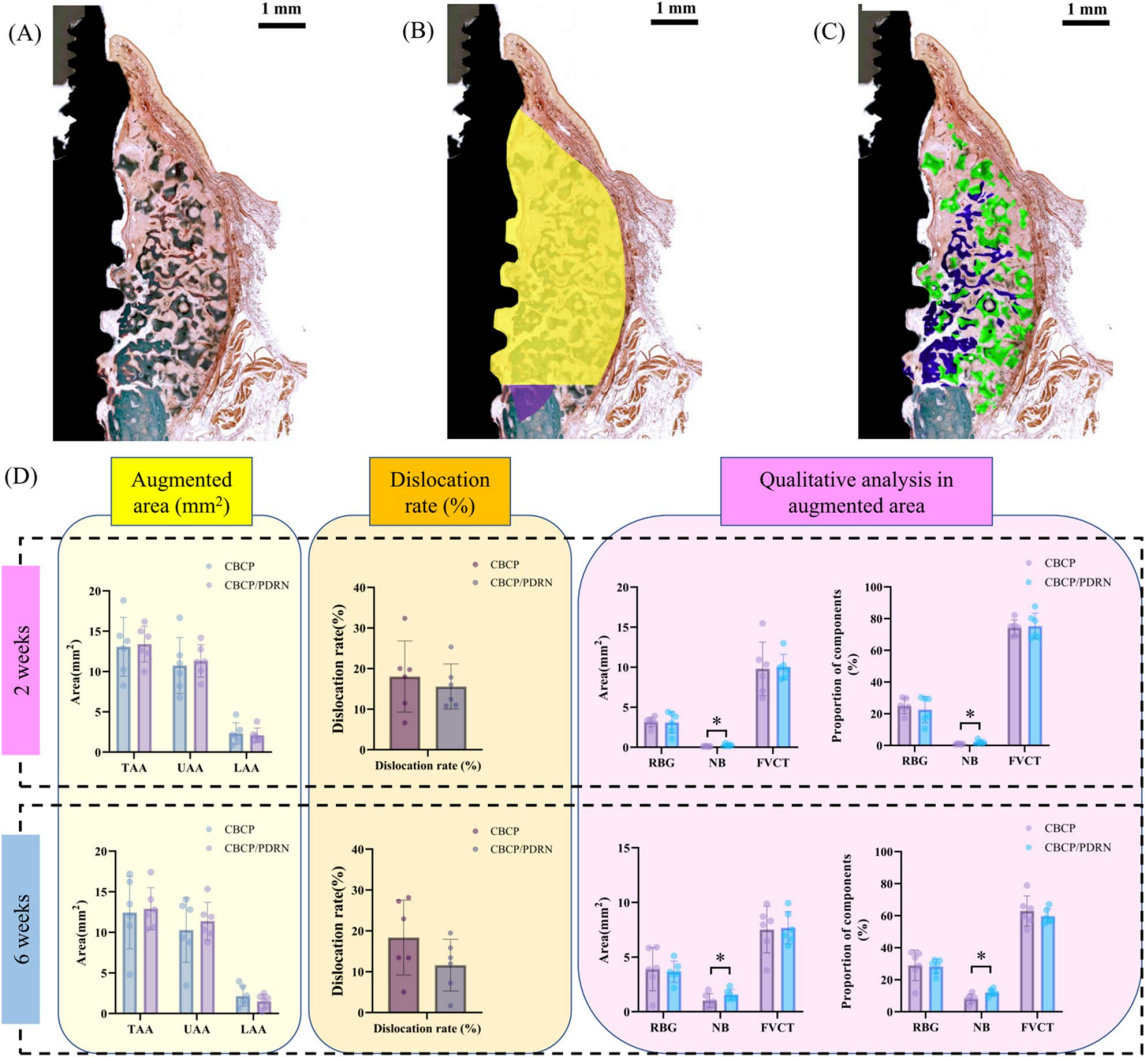
Histomorphometric analysis in the augmented area. (**A**) The augmented area is composed of new bone (NB), residual bone graft (RBG), and fibrovascular connective tissue (FVCT). (**B**) The total augmented area (TAA) was divided into the upper augmented area (UAA, yellow) and the lower augmented area (LAA, purple). The dislocation rate was calculated by LAA/TAA (%). (**C**) NB and RBG were demarcated with blue and green colors, respectively. (**D**) The total augmented area (mm^2^) and dislocation rate (%) were measured in the qualitative analysis of the augmented area. New bone (NB) in the CBCP/PDRN group was significantly higher, both in terms of area and proportion at 2 and 6 weeks, than in the CBCP group.

The dislocation rate and related parameters in the augmented area are presented in Fig. 6. At 2 weeks of observation, TAA was 13.07 ± 3.66 mm^2^ in the CBCP group and 13.41 ± 2.22 mm^2^ in the CBCP/PDRN group (p = 0.8504). UAA was 10.74 ± 3.46 mm^2^ in the CBCP group and 11.31 ± 2.01 mm^2^ in the CBCP/PDRN group (p = 0.7211). LAA was 2.33 ± 1.32 mm^2^ in the CBCP group and 2.09 ± 0.89 mm^2^ in the CBCP/PDRN group (p = 0.6713). The dislocation rate was 18.04 ± 8.77% in the CBCP group and 15.59 ± 5.55% in the CBCP/PDRN group, demonstrating no statistically significant difference between the groups (p = 0.4468).

In the observations at 6 weeks, TAA was 12.44 ± 4.45 mm^2^ in the CBCP group and 12.91 ± 2.60 mm^2^ in the CBCP/PDRN group (p = 0.8272). UAA was 10.29 ± 3.98 mm^2^ in the CBCP group and 11.39 ± 2.32 mm^2^ in the CBCP/PDRN group (p = 0.5695). LAA was 2.15 ± 1.25 mm^2^ in the CBCP group and 1.52 ± 0.86 mm^2^ in the CBCP/PDRN group (p = 0.3299). The dislocation rate was 18.37 ± 9.19% in the CBCP group and 11.61 ± 6.34% in the CBCP/PDRN group, demonstrating no statistically significant difference between the groups (p = 0.1689).

#### Qualitative analysis in the augmented area

The outcomes of the qualitative analysis in the buccal augmented area are presented in Fig. 6. At 2 weeks, the RBG area was 3.15 ± 0.60 mm^2^ in the CBCP group and 3.11 ± 1.32 mm^2^ in the CBCP/PDRN group (p = 0.9368). The RBG proportion was 24.95 ± 4.86% in the CBCP group and 22.59 ± 8.27% in the CBCP/PDRN group (p = 0.6176). The NB area was 0.11 ± 0.05 mm^2^ in the CBCP group and 1.27 ± 0.15 mm^2^ in the CBCP/PDRN group; this value showed a statistically significant difference between the two groups (p = 0.0281). The NB proportion was 0.86 ± 0.34% in the CBCP group and 2.08 ± 1.19% in the CBCP/PDRN group, which likewise presented a statistically significant difference between the two groups (p = 0.0319). The FVCT area was 9.80 ± 3.34 mm^2^ in the CBCP group and 10.03 ± 1.60 mm^2^ in the CBCP/PDRN group (p = 0.8957). The FVCT proportion was 74.19 ± 4.99% in the CBCP group and 75.32 ± 8.14% in the CBCP/PDRN group (p = 0.8089).

In the 6-week observations, the RBG area was 3.89 ± 1.97 mm^2^ in the CBCP group and 3.66 ± 0.96 mm^2^ in the CBCP/PDRN group (p = 0.8021). The RBG proportion was 29.00 ± 9.54% in the CBCP group and 28.25 ± 4.47% in the CBCP/PDRN group (p = 0.8651). The NB area was 1.03 ± 0.63 mm^2^ in the CBCP group and 1.56 ± 0.47 mm^2^ in the CBCP/PDRN group, and this value showed a statistically significant difference between the groups (p = 0.0131). The NB proportion was 8.07 ± 3.01% in the CBCP group and 12.04 ± 2.11% in the CBCP/PDRN group, which also constituted a statistically significant difference (p = 0.0247). The FVCT area was 7.52 ± 2.14 mm^2^ in the CBCP group and 7.68 ± 1.48 mm^2^ in the CBCP/PDRN group (p = 0.8387). The FVCT proportion was 62.93 ± 9.43% in the CBCP group and 59.72 ± 4.60% in the CBCP/PDRN group (p = 0.3547).

## Discussion

3D-printed implant has been introduced recently because it could provide similar biological properties to conventional implants with cheap^29^. The development of cone beam computed tomography, oral scanning, and computer-aided design software could also contribute to the increase and accuracy of 3D-printed implant. However, 3D-printed implant installation into dehiscence defect with bone regeneration procedure was not fully investigated. Dehiscence defects can be encountered at the time of implant placement. If not corrected, dehiscence defects can be a risk factor for peri-implant disease during long-term follow-up and can cause aesthetic problems due to peri-implant mucosal recession^30^. Simultaneous bone augmentation at the time of implant placement has been considered as a predictable treatment modality as reported in preclinical and clinical studies^31,32^. In terms of alveolar ridge dimensions, regenerative procedures for correcting minor peri-implant bone defects showed less contraction of the alveolar ridge width than when a regenerative procedure was not performed, improving esthetic outcomes^33^. The validity of bone augmentation with implant placement was also observed for the type of 3D-printed implant used in this study, as shown by the increased BIC and BAFO in the buccal augmented area, irrespective of PDRN application (Fig. 5).

The present preclinical study demonstrated that peri-implant new bone formation was improved in the CBCP/PDRN group compared to the CBCP group in buccal augmented sites in a dehiscence defect model. The study showed that (i) there were no differences between the CBCB and CBCB/PDRN groups in terms of the parameters analyzed using micro-CT; (ii) the NB area (mm^2^) and proportion of NB in augmented area could be expected to improve at 2 and 6 weeks using CBCP/PDRN; (iii) the TAA (mm^2^) using CBCP was not significantly affected by PDRN; and (iv) the dislocation rate was approximately 15%, without statistical differences between the two CBCP and CBCP/PDRN groups.

The micro-CT analysis revealed no significant differences in parameters, including BV/TV, BS/BV, Tb.Pf, and SMI, between the CBCP and CBCP/PDRN groups. However, the CBCP/PDRN group showed higher NB area (mm^2^) and NB proportion (%) values in the buccal augmented area. This discrepancy seems to be attributable to the expanded volume of interest in the micro-CT analysis. PDRN was applied only to the buccal augmented area; therefore, the effect of PDRN was confined to the buccal augmented area and did not extend to the non-augmented area. An explanation for the lack of a difference between the CBCP and CBCP/PDRN groups in the parameters obtained from the micro-CT analysis may be that the augmented area was much smaller than the non-augmented area.

The NB area (mm^2^) and proportion (%) was higher in the CBCP/PDRN group than in the CBCP group. Various studies have demonstrated the biological activity of PDRN, including angiogenesis, anti-inflammatory effects, the enhancement of collagen synthesis, and the facilitation of osteoblast activity. NB formation can be upregulated by angiogenesis, which brings precursors, oxygen, and nutrients to metabolically regenerating tissue^10^. Enhanced osteoblast activity can also lead to early bone formation^34^. The increase in NB in the CBCP/PDRN group can be attributed to these effects, and the results suggest that PDRN may be applicable as a treatment modality for early bone formation in buccal dehiscence defects. Our previous study applied PDRN in maxillary sinus floor elevation procedures and confirmed that bone regeneration was promoted in the upper part of the sinus, which has lower osteogenic potential than near the pristine bone^15^.

The NB area (mm^2^) and proportion (%) from the histomorphometric analysis showed significantly higher values in the CBCP/PDRN group than in the CBCP group. However, BIC and BAFO failed to show corresponding outcomes, possibly due to the observation time points. This study only investigated healing at 2 and 6 weeks to determine the radiographic and histological results of early bone formation. These histomorphometric outcomes might change in a healing period of more than 12 weeks, which would include the bone remodeling process. In the remodeling process, the differences between the two groups in BIC or BAFO might become significant.

In this study, TAA in the buccal augmented area remained consistent over the experimental period. The soft block type of collagenated bone showed space maintenance (approximately 13 mm^2^) until 6 weeks in this study. Previous studies have shown that alveolar bone reconstruction with the soft block type of collagenated bone led to predictable outcomes^16–18^. The soft block type of collagenated bone showed improvements in stability and reduced the displacement of bone graft material, with a dislocation rate of approximately 15% in this study. Despite some differences in measurement methods, a prior study reported that the displacement of bone graft material during the suturing procedure appeared up to about 44% when particle-type bone graft material was used^35^. However, block-type bone graft material showed less displacement. Since the stability of the carrier applied with biologically active PDRN has a very critical effect on the biological response, the block type of collagenated bone showed its potential as a carrier. Several studies have also demonstrated the potential of collagenated block bone as a carrier for various growth factors or bioactive agents, such as collagen matrix^21–23^.

The limitation of our study is that it did not include longer healing period and the diverse carrier system of PDRN. Although collagenated bone substitute could be used as carrier for PDRN, another scaffold could be used to deliver PDRN considering releasing kinetics^12^. Second, the buccal dehiscence defect was created intentionally standardized size for analysis, resulting in exposed trabecular bone. In clinical situation, most dehiscence defect is found due to the insufficient bone width and the nature is cortical bone. The healing pattern of trabecular bone might be different from that of cortical bone. The healing pattern of dehiscence defect with cortical bone with chronic defect model is needed. Finally, the efficacy and safety of PDRN in hard tissue regeneration should be investigated in human in future study.

In summary, peri-implant buccal dehiscence defects immediately followed by 3D-printed implant placement were corrected with a collagen membrane and CBCP or CBCP/PDRN. As parameters obtained from the micro-CT analysis, BIC and BAFO showed slight differences between the CBCP and CBCP/PDRN groups. However, the CBCP/PDRN group showed improvements in the NB area and NB proportion in the buccal augmented area. In addition, the use of PDRN might have the potential to facilitate early bone formation, with sufficient stability over time, in dehiscence defects.

## Data Availability

The data that support the findings of this study are available from the corresponding author upon reasonable request.

## Abbreviations

PDRN: Polydeoxyribonucleotide
CBCP: Collagenated biphasic calcium phosphate
VOI: Volume of interest
BV: Bone volume
TV: Tissue volume
BS: Bone surface
Tb.Pf: Trabecular bone pattern factor
SMI: Structure model index
BIC: Bone to implant contact
BAFO: Bone area fraction occupancy
NB: New bone
RBG: Residual bone graft
FVCT: Fibrovascular connective tissue
TAA: Total augmented area
UAA: Upper augmented area
LAA: Lower augmented area
